# Implementing a community model of early pregnancy care

**DOI:** 10.1186/s12913-020-05524-8

**Published:** 2020-07-17

**Authors:** Rebecca Cox, Somia Khalid, Gemma Brierly, Annie Forsyth, Ruth McNamara, Victoria Heppell, Ingrid Granne

**Affiliations:** 1 Nuffield Department of Primary Care Health Science, Oxford, UK; 2 Oxford University Hospitals NHS Foundation Trust, Oxford, UK

**Keywords:** Early pregnancy, Community care, Pregnancy loss, Safety, Efficacy, Patient feedback

## Abstract

**Background:**

In the UK Early Pregnancy Assessment Units (EPAUs) are usually situated alongside hospital maternity and gynaecology services. In June 2018, the Oxford EPAU relocated from the John Radcliffe Hospital to a community clinic. This is to our knowledge, the UK’s first community-based EPAU. This change was inspired by our patient feedback describing the co-location of the EPAU with maternity services as distressing.

**Methods:**

Following the introduction of the community EPAU we developed a database to capture information on the patients seen in the clinic. This is a retrospective observational study of a single cohort of patients attending the clinic over an 8 month period. Data was collected from 1st July 2018 to 28th February 2019. This data included clinical, safety and patient experience outcomes.

**Results:**

Two thousand nine hundred and twenty patient episodes were recorded, 1,932 were new patients. Mean waiting time to be seen in clinic was 1.3 days. When miscarriage was confirmed 48.6% chose conservative management, 19.9% chose medical management, and 31.5% chose surgical management. The mean rate of ambulance transfers to hospital was 3.1 per month. Of all patients seen in EPAU 32 had unplanned admissions, which accounted for 2.7% of all patients seen in EPAU. Patient feedback questionnaires have been consistently positive.

**Conclusion:**

The development of a community EPAU has improved services to allow care closer to home in an environment separate from maternity care. Our data shows that a community EPAU can deliver timely, good quality patient care, is safe, and a service valued by patients. Further research is indicated to evaluate the cost-effectiveness of community EPAUs and the long term safety and effectiveness of care.

## Background

Symptoms of pain and bleeding in early pregnancy are common, occurring in 20–40% of pregnancies [1] and account for a significant proportion of medical care in pregnancy. Miscarriage occurs in around 20% of all pregnancies and ectopic pregnancy, which can be life threatening, affects 2% of pregnancies [1]. About 125,000 miscarriages occur annually in the United Kingdom [1] and account for over 42,000 hospital admissions each year [1]. In addition to the physical symptoms, experiencing miscarriage can be an extremely distressing and emotional time, sometimes involving multiple hospital visits, alongside medical or surgical treatment.

The Early Pregnancy Assessment Units (EPAU) as a specialist service is a concept that has been in gynaecology care for more than 20 years. EPAUs were developed to offer better care to women and reduce admissions to gynaecology wards, and are usually located within obstetric and gynaecology departments facilitating communication with acute gynaecology care. International evidence from Canada and Australia has shown that EPAUs facilitate reduced length of stay in A&E, reduced hospital admissions, and reduced unplanned admissions [2, 3].

Due to this co-location with obstetric and gynaecology services patients attending the EPAU will very commonly come into contact with pregnant ante-natal patients in order to access the EPAU, despite themselves losing their pregnancy. Unsurprisingly, a common feature of patient complaints, blogs and forums is that this colocalisation lacks sensitivity and compassion and can significantly heighten the stress of their situation . There is a large variation in the way EPAU services run across the UK, with differences in levels of care, accessibility and staffing. The VESPA study [4], is currently being conducted to investigate these variations and their effects on clinical, service and patient-centred outcomes [4].

In June 2018, Oxford EPAU relocated from the John Radcliffe Hospital to a community clinic: to our knowledge the UK’s first community-based EPAU. This change was inspired firstly by local patient feedback describing co-location of an EPAU with maternity services as problematic. Secondly, the move is in keeping with the NHS 5-Year Forward View, advocating extending care outside of hospital to allow easier access for patients and relieve the pressures on acute hospitals. Patients and other stakeholders were integral to the service development and implementation of the clinic.

This relocation was made possible by technological advances and developing a highly trained specialist core nursing team. The use of point of care blood HCG testing, facilitated rapid clinical decision making in the management of pregnancy of unknown location (where a patient has a positive pregnancy test but there is no evidence of an intra or extra uterine pregnancy on ultrasound) without the need for a hospital laboratory in the majority of cases. The development of electronic patient records means that clinical notes can be accessed in the community or within the hospital setting, should they be required, to allow seamless patient care.

The community clinic has been operating for 1 year, running a service that provides care for pregnant women who experience problems in early pregnancy up to 16 weeks gestation. Patients can self-refer to the clinic, receive ultrasound scans, and the subsequent necessary care without going to hospital. In cases requiring urgent hospital admission the ambulance service is utilised. In developing new services such as a community EPAU it is essential to ensure and demonstrate that the service is safe, effective, and acceptable to patients.

## Methods

### Aims

- To assess the clinical performance, safety, and patient feedback of the Oxford Community EPAU in key outcomes:
- Clinical outcomes: referral rate to hospital, patients receiving conservative management of miscarriage, ruptured ectopic pregnancies, negative ectopic laparoscopies, unplanned admissions to hospital.
- Safety outcomes: unplanned admissions to hospital and the number of ambulance transfers to hospital.
- Patient experience outcomes: patient feedback in quality of care, overall experience, nursing care, and environment.

### Design

This study is an observational study of a single cohort of patients attending the Oxford Community EPAU. It includes a eight-month retrospective review of fully anonymised clinical and safety data collected from the clinic, from the 1st July 2018 to 28th February 2019. Prospective patient feedback data was collected in surveys given to patients from this period.

### Location

The clinic is a NHS Early Pregnancy Assessment clinic located in the community in Oxford, UK.

### Participants

The study included all patients who were less that 16 weeks gestation who had attended the clinic between these dates. Common reasons that patients attend the clinic are pain or bleeding in early pregnancy. Patients could self-refer into the clinic, be referred by A&E, or by their GP. The study included patients who were attending for follow-up appointments and repeat ultrasound scans. Patients who attended the clinic then were admitted to hospital were followed up through hospital records.

### Data collection

All patients who attend the Oxford EPAU are prospectively recorded in a database. This anonymised database records reasons for referral, symptoms, investigation results and clinical outcomes. This database was reviewed retrospectively to identify and record the clinical outcomes of this study. Table 1 shows the list of variables collected and the sources. Data was collected on patient demographics, gestation, presenting symptoms, diagnosis, clinical management, referral source, referral numbers, unplanned admissions, ambulance transfers, and adverse events.

**Table 1:** Data collected and sources

Data	Source
Patient demographic data	EPAU database
Gestation	EPAU database
Presenting symptoms	EPAU database
Referral source	EPAU database
New patient episode or review	EPAU database
Diagnosis	EPAU database
Management	EPAU database
Ectopic patient data inc. diagnosis, ultrasound results, management, and surgical results.	Electronic patient record, electronic ultrasound records, electronic surgical records
Referral to inpatient gynaecology ward	EPAU database
Ambulance transfers	Serious event folder, South Central Ambulance
Unplanned admission data	Gynaecology ward database
Adverse patient outcomes	Clinical governance records

Patient feedback was collected through a self-designed paper survey that was given to patients in the unit (additional file 1). The survey contained 13 questions, 11 of which were on a numerical rating scale, with 2 free-text feedback questions. The survey was designed to capture information on experience, staff, environment, and quality of care. Every patient who attended the clinic was given the opportunity to do the questionnaire, which was completed in the clinic and put in a sealed box at the reception desk. The surveys were then added to a database by the EPAU nursing team.

### Bias

This is an observational study using a single cohort of patients for clinical performance and safety. All new patients who came to the clinic were included and the data for all patients was analysed by the same researcher anonymously. For the comparison of patient feedback between hospital and community, data was collected anonymously using the same questionnaire placed by the patient into a feedback box, and assessed anonymously by one researcher.

### Statistical analysis

Descriptive analysis of the data collected from the clinic was performed using Microsoft Excel to assess the outcomes investigated. There were no concerns regarding missing data as all data was available from the clinic.

## Results

Over the 8 month period from 1st July 2018 to 28th February 2019 2920 patient episodes were recorded, including patients attending as initial new referrals and follow up appointments. One thousand nine hundred thirty-two of these episodes were new patients and 988 were returning patient episodes.

The mean age of patients presenting to the clinic was 31.1 years (range of 13 to 51 years).

The mean gestation of pregnancy gestation was 8.1 weeks (range 4–16 weeks).

### Waiting time

The mean waiting time from referral to being seen for an appointment in clinic was 1.3 days (range 0–14). Patients were seen on the same day for urgent referrals, and the longest wait times were 2 weeks to comply with clinic protocols for some patients requiring repeat scans.

### Referral source

Referrals were classified into self, GP, A&E, and gynaecology ward referrals. 58.8% of referrals were self- referrals by patients. GPs made 20.3% of referrals, the hospital gynaecology ward referred 7.5%, and A&E made 3.8% of referrals. Other sources contributed 9.6% of referrals, which were from midwives, antenatal radiology clinic, and from local termination and fertility services.

### Presentation

The commonest initial presentation to EPAU was with bleeding 43.5%, with pain 19.8%, both bleeding and pain 22.5, and 14.2% for other reasons such as previous ectopic pregnancy.

### Referrals from EPAU clinic to hospital

Data was collected on the number of patients referred onwards from the EPAU into hospital. During the 8 month period a total of 113 patients were referred into hospital. Reasons for further referral were suspected ectopic pregnancy, diagnosed ectopic, heavy bleeding, severe pain, molar pregnancy, and for the management of retained products of conception (see Additional file 2). One patient was referred for urgent removal of intrauterine coil. Elective referrals for surgical management are not included in this data.

### Diagnosis

Following clinic attendance 72.2% (*n* = 1045) of patients were diagnosed with a viable intrauterine pregnancy. 389 (*n* = 389) miscarriages were diagnosed, of these 84.1% (*n* = 327) were missed or incomplete miscarriages, 15.9% (*n* = 62) were complete miscarriages (see Additional file 3).

### Management of confirmed miscarriage

We reviewed each diagnosed miscarriage from the EPAU to find out what type of management the patient chose after the initial diagnosis. 48.6% (*n* = 159) chose to have conservative management, 19.9% (*n* = 65) chose medical management, and 31.5% (*n* = 103) chose surgical management.

### Ambulance transfers

**Fig. 1:**
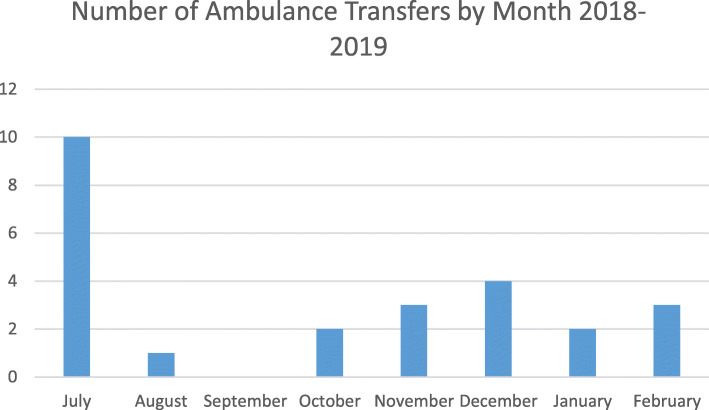
Graph ambulance transfers by month

During July 2018 to February 2019 there were 25 ambulance transfers from EPAU to the John Radcliffe Hospital. The mean rate of transfers per month was 3.1 (range 0–10). The highest figure of ten was found in July 2018, the first month of the community clinic, then falls to a steady state between one and four per month (Fig. 1).

Six patients were transferred because a ruptured ectopic pregnancy was diagnosed on ultrasound. Four stable patients with suspected ectopic pregnancies or bleeding were transferred by ambulance as they were alone with no transport to get to hospital. Eight patients were transferred due to ectopic or suspected ectopic diagnosis. Seven patients were transferred for heavy bleeding or collapse with a diagnosis of miscarriage or PUL. These figures exclude stable ectopic patients as the ambulance transfer protocol allows them to be driven to hospital if accompanied.

### Unplanned admissions

Unplanned admission of patients seen in the community EPAU was collected for the months December 2018–April 2019. During this time there were 32 admissions to gynaecology ward of patients who had been seen or in contact with EPAU and had either self referred to the ward or attended via A&E. These account for 2.7% of all patients seen in EPAU during this time period. Three referrals were self referrals, two were from GPs, and one was from paramedics, the remaining 24 were from A&E. 23 (71.8%) of the patients were patients seen in EPAU and then developed worsening symptoms of pain and bleeding so attended A&E or the gynaecology ward. Three patients booked appointments for EPAU but attended A&E/gynaecology ward due to symptoms before appointment. Two patients were referred to secondary care by their GP after the patient spoke to EPAU. Ten patients who had not been to the EPAU presented to A&E during this time period with an ectopic pregnancy.

### Ectopic pregnancy outcomes

**Fig. 2:**
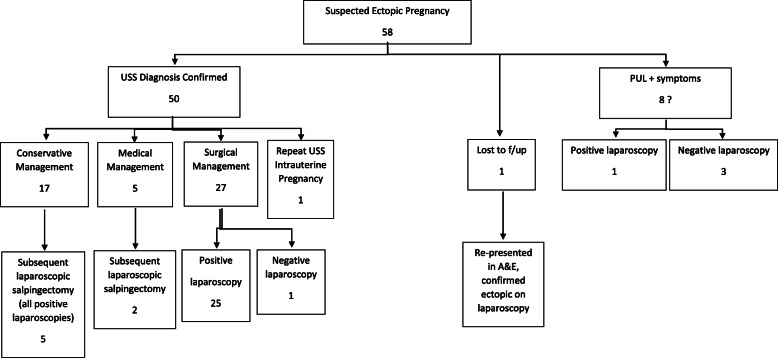
Flow diagram of ectopic pregnancy outcomes

Fifty eight patients had ultrasonographically confirmed or suspected ectopic pregnancies out of 1932 new patients (3%). Seventeen of these patients were managed conservatively (BHCG< 1000), of those five went on to have surgical management of non-ruptured ectopic pregnancies (Fig. 2).

Five patients who met local guidelines for medical management were treated with methotrexate. Two of these patients went on to have surgical management- one of which was a suspected ectopic and operated on out of hours.

Twenty seven patients were managed surgically with laparoscopic salpingectomy once confirmed. One patient had a negative laparoscopy (3.7%).

Eight patients had no evidence of intrauterine or extrauterine pregnancy on ultrasound scan, but based on clinical symptoms went on to have laparoscopy. Three of these patients had a negative laparoscopy.

### Patient feedback data

Eighty five patient feedback surveys were collected in the year that the new clinic opened. We also collected 40 surveys from the hospital based EPAU clinic prior to moving location. In the community clinic quality of care rated excellent increased from 60 to 89%, emotional support rated excellent improved from 50 to 74.7%, and overall experience rated excellent improved from 57.5 to 81.7%. The average waiting time improved from 65% seen within 1–2 days and none seen same day, to 70.7% seen in 1–2 days and 9.8% seen same day.

## Discussion

### Effectiveness of the clinic

The NHS 5 year forward view advocates care closer to home, which this clinic aims to provide. The average waiting time to be seen in clinic was 1.3 days, reflecting patients being seen in a timely manner. More than half of patients (58.8%) were self-referrals, increasing access to clinic. The most common reason for referral was bleeding in early pregnancy. Of the patients attending the clinic 72.2% were viable intrauterine pregnancies. From the miscarriage group 84.1% were missed or incomplete miscarriages, 15.9% were complete miscarriages. First choice of management of miscarriage was most commonly conservative management 48.6%, followed by 31.5% choosing surgical management and 19.9% choosing medical management. The high percentage of conservative management reflects the role of the specialist nursing team, bedside technology with electronic patient records and point of care testing encourages patients to feel comfortable in the community, avoiding the need for hospital management. NICE 2019 recommends conservative management as first choice for first trimester miscarriages [5]. The 2006 MIST trial showed that expectant or medical management of miscarriage produced significantly more unplanned hospital admissions compared to surgical management [6], however the emotional wellbeing and impact of personal choice on management are important factors when designing services. Our service offering medical and expectant management has shown excellent patient feedback and a majority of patients choosing expectant care.

Three studies have shown the introduction of an EPAU resulted in a shorter length of stay in both emergency departments [3] and outpatient clinics [2], a reduction in the proportion of women requiring hospital admission [2, 3] and a reduction in the number of women representing to health services [3]. Our review has shown low numbers of unplanned admissions and very few ambulance transfers when required to secondary care.

From the patient feedback data collected, patients consistently rated the community clinic higher than the previous hospital clinic, covering areas such as quality of care, environment, staff and emotional support. A 2009 UK study found that over 80% of women rated their satisfaction with privacy, dignity, and care as excellent [7]. There is minimal published data on this area in EPAUs, but our results are very positive.

Patients were directly triaged on the telephone from self-referral by a senior nurse, who could appropriately decide if the patient met criteria to come to the EPAU or direct referral to hospital. The low numbers of referrals to A&E indicate this triage is likely effective. From the ectopic pregnancy data the majority of patients received surgical management. From this group of patients there was a low negative laparoscopy rate of one patient, a rate of 3.7%, compared to a rate of 6% in a 2016 study [8]. There is little existing data on negative laparoscopic rates, but it is reassuring to find that our rate is lower than the existing literature. Three patients had negative laparoscopies but these were investigative procedures due to clinical findings with a background of miscarriage or pregnancy of unknown location. From the group of ectopic pregnancies managed conservatively or medically, 6 went on to have surgical treatment, a rate of 31.6%.

### Safety of clinic

The clinic provides highly trained nurse sonographers and nursing staff who have received skills, drills and human factors training. During the eight months of data collection 25 ambulance transfers were made from the clinic to the John Radcliffe hospital, four of these were for patients who had no transport themselves, and the remaining patients were all due to medical emergency such as being haemodynamically unstable, bleeding, pain, faint, or ruptured ectopic. The use of ambulance transfers was decided based on rigid criteria for patient safety based on the ectopic pregnancy protocol established in the clinic. The typical cost of an ambulance transfer is approximately £250. The first month working in the new community setting there were 10  transfers, however this reduced to 1–4 per month as staff became more confident in assessment. The low number of transfers per month reflects the appropriate triage of the nursing team upon referral, patients with significant pain, heavy bleeding would be triaged to secondary care. Emergency admissions to hospital from EPAU made up only 1.8% of patients seen in the community EPAU. The 2006 MIST trial was a seven centre randomised controlled trial looking at infection and unplanned admission outcomes from conservative, medical and surgical treatments for miscarriage. Compared to the MIST trial our unplanned admission rate is low, the MIST trial reports 49% of conservative management patients, 8% surgical management patients, and 18% of medical management patients having unplanned admissions [6]. The MIST trial did not publish their overall unplanned admission rate for all types of management. 71.8% of patients that were unplanned admission to hospital were due to worsening symptoms after attending EPAU, this can be expected as the risks of conservative and medical management of miscarriage include worsening pain or bleeding, which patients are warned about and safety netted to call the gynaecology ward or A&E. It is difficult to compare unplanned admission rates between centres, as there will be differences in populations, protocols, and services provided; however our study shows that our unplanned admission rates are better.

### Generalisability

Our data is from a single clinic in Oxford, despite this it includes all new patients that presented to the clinic in an 8 month period, this includes a wide variety of patients from diverse backgrounds, and would be comparable to other centres in the UK looking after patients in early pregnancy.

### Limitations of study

The data collected in this evaluation was based on a prospective clinical database with information entered by clinic staff. There was a risk of data being entered inaccurately. To reduce the error form this we crossed checked data with electronic patient records and surgical records. The data was collected during the first 8 months of the clinic opening, hence may not represent the performance of the clinic if more time had lapsed to allow the clinic to settle into day to day running. A repeat collection of this data in 1 year would be helpful to identify any changes. The data collected on patient feedback was based on a survey design. This could be improved using formal focus group and interview techniques to collect high quality qualitative data. Bias may be present in the patient feedback, with patients preferably rating services in a new modern clinic compared to an older existing service.

## Conclusion

### Main conclusions

The development of a community EPAU has improved services to allow care closer to home in an environment separate from maternity care for women experiencing pregnancy loss. Patients have been seen in a timely manner and given choice of their own management for pregnancy loss. The clinic provides highly trained staff able to triage safely, evidenced by low emergency transfers to hospital. The low negative laparoscopy rates indicate safety and accuracy of clinical diagnosis in the clinic.

Further research is indicated to evaluate the cost effectiveness of community EPAUs and long term safety and effectiveness of care.

## Supplementary information

**Additional file 1.** Emergency Clinic Questionnaire.

**Additional file 2.** Flow diagram referrals to hospital.

**Additional file 3.** Flow diagram initial appointments.

## Data Availability

The datasets used and/or analysed during the current study are available from the corresponding author on reasonable request.

## Abbreviations

A&E: Accident and emergency department
EPAU: Early pregnancy assessment unit
HCG: Human chorionic gonadotrophin
NICE: National Institute of Clinical Excellence
PUL: Pregnancy of unknown location
