# Supplementary material for: Implementing a community model of early pregnancy care
Source: BMC Health Serv Res. 2020 Jul 17;20:664. doi: 10.1186/s12913-020-05524-8 (PMC7367246; doi:10.1186/s12913-020-05524-8)
Supplement: Supplementary file 1 — Additional file 1. Emergency Clinic Questionnaire. [file 12913_2020_5524_MOESM1_ESM.docx]

*Emergency Clinic Questionnaire*

We hope that the clinic today offered you a caring and supportive appointment. We are always looking for ways to improve the clinic for future women and would be grateful if you could spare a few minutes to complete this questionnaire:

How long did you wait for your appointment?

1. 1-2 days B)2-3 days C) 3-4 days D) longer: days

Were you given enough privacy when during the consultation and examination?

1. Excellent b) very good c) good d)satisfactory e)unsatisfactory f)poor

Were the staff friendly and welcoming?

1. Excellent b) very good c) good d)satisfactory e)unsatisfactory f)poor

Did you find your consultation:

1. Helpful yes/no b) sensitive yes/no c) informative yes/no

Were you involved with decisions about your care?

A) Yes B) No

When you had important questions to ask a health professional, did you get answers that you could understand?

A) Yes B) No

How would you rate the quality of care?

1. Excellent b) very good c) good d)satisfactory e)unsatisfactory f)poor

How would you rate the level of emotional support given?

1. Excellent b) very good c) good d)satisfactory e)unsatisfactory f)poor

Did you feel you were treated with respect and dignity while you were in the hospital?

A) Yes B) No

Were you happy with the information leaflets given?

1. Yes b) No

Overall experience

1. Excellent b) very good c) good d)satisfactory e)unsatisfactory f)poor

What are we doing well:

What do we need to improve:

Comments about the facilities, including the building and waiting area:
