# Supplementary material for: Implementing a community model of early pregnancy care
Source: BMC Health Serv Res. 2020 Jul 17;20:664. doi: 10.1186/s12913-020-05524-8 (PMC7367246; doi:10.1186/s12913-020-05524-8)
Supplement: Supplementary file 2 — Additional file 2. Flow diagram referrals to hospital. [file 12913_2020_5524_MOESM2_ESM.docx]

Referrals to Hospital

77 (3.9% total new patients seen)

Incomplete miscarriage for surgery

3

Retained products conception

8

Confirmed ectopic

16

PUL + symptoms

7

7

Molar

2

Heavy bleeding or pain

7

Other

7

Suspected ectopic

27
