# Supplementary material for: Implementing a community model of early pregnancy care
Source: BMC Health Serv Res. 2020 Jul 17;20:664. doi: 10.1186/s12913-020-05524-8 (PMC7367246; doi:10.1186/s12913-020-05524-8)
Supplement: Supplementary file 3 — Additional file 3. Flow diagram initial appointments. [file 12913_2020_5524_MOESM3_ESM.docx]

Complete

62

Initial patient choice

Surgical management

103

Medical management

65

Conservative management

159

Pregnancy Unknown Location

200

Suspected/confirmed ectopic

59

Intrauterine Pregnancy Uncertain Viability

Discharged

4

Discharged

17

BHCG

3

Repeat USS

337

Repeat USS

7

Ward referral

6

BHCG

178

Miscarriage

389

Discharged

1045

Viable Intrauterine Pregnancy

1045

New Patient Episodes

1932
